# Magnesium—An Ion with Multiple Invaluable Actions, Often Insufficiently Supplied: From In Vitro to Clinical Research

**DOI:** 10.3390/nu15143135

**Published:** 2023-07-13

**Authors:** Mario Barbagallo, Nicola Veronese, Ligia J. Dominguez

**Affiliations:** 1 Geriatric Unit, Department of Medicine, University of Palermo, 90100 Palermo, Italy; nicola.veronese@unipa.it; 2 Faculty of Medicine and Surgery, University Kore of Enna, 94100 Enna, Italy; ligia.dominguez@unikore.it

Magnesium (Mg) is a key ion for numerous metabolic processes, being a cofactor of over 600 enzymes involved in cell metabolism and multiple biological processes. Mg is needed for mitochondrial adenosine triphosphate (ATP) synthesis and energy production, nucleic acid synthesis and stability, protein synthesis, as well as oxidative phosphorylation. Mg is also involved in the action of glycolytic enzymes, protein kinases, and all phosphorylation processes and reactions that implicate ATP consumption. Globally, around 70% of the body’s enzymes require Mg to function properly [1].

The total content of Mg in the human body is around 24 g (1 mole) and it is mostly intracellular. Mg is a major component of bone, together with calcium (Ca), while only 1% of Mg is present in the blood. Serum Mg concentrations are strictly controlled with a normal range between 0.75 and 0.95 mmol/L (1.7–2.5 mg/dL or 1.5–1.9 meq/L). Because of this tight control, total serum Mg concentrations (MgT) do not always accurately reflect the body’s Mg status. Hypomagnesemia (defined as a serum Mg level below 0.75 mmol/L) occurs only with a severe Mg deficiency, while the majority of subclinical Mg deficiency remains undetected. Thus, MgT is rarely helpful in identifying subclinical Mg deficits. A Mg-to-Ca ratio may be useful since functions of both cations are closely linked. Thus, Mg is a mild Ca antagonist, and the cellular influx/efflux of Mg and Ca use the same transporters [2].

Although there is no agreement among the different national and international guidelines, the amount of dietary Mg required is suggested to be around 300–400 mg per day in healthy adults (5 to 6 mg/kg/day). Mg is the central core of the chlorophyll molecule in plant tissue; thus, it is present in all green leafy vegetables. Although Mg is present in many other foods, including legumes, seeds, nuts, seafood, chocolate, and whole versus refined grains, Western diets lacking these types of foods are generally low in Mg. Nutritional Mg insufficiency has been frequently found in Western industrialized countries. Data from the NHANES III in the US and from France showed that Mg intake in the population of all ages is well below the recommended daily requirement [3,4]. This corresponds with the low consumption of foods rich in Mg reported by the Dietary Guidelines for Americans 2015–2020 [5] (% of persons below recommendation across all ages and both sexes in the US was near 100% for whole grains, ~90% for total vegetables, over 80% for beans and peas, and ~60% for nuts, according to data from NHANES 2007–2010), which also indicated that 49% of the US population, considering all age groups, had a Mg intake below the estimated average requirement. Other estimates have indicated that over 60% of Americans are under the recommended daily intake [6].

Processed and ultra-processed foods that constitute a substantial portion of the diet in Western countries have a significantly reduced Mg content, and a significant proportion of this ion (up to ~80%) is lost during food processing, cooking, or refining [7].

In agriculture, soil acidification has become a major global issue. The progressive acidification of soils in the last fifty years has mainly been caused by the release of protons (H^+^) during the transformation and cycling of carbon, nitrogen, and sulfur and fertilizer reactions. Soil acidification may have contributed to Mg scarcity since reduced Mg levels have been associated with acid soils. Some pesticide agents employed in agriculture in crops, such as glyphosate, chelate Mg [8], further decreasing the content of Mg in the soil. In relation to this, it has also been suggested that the Mg content in vegetables and fruits has dropped in recent years.

The benefits of Mg are known in terms of gut motility and gastric acidity, although recent evidence has suggested that dietary Mg may also positively affect gut microbiota [9] and be a key player in inflammatory bowel disease [10]. Because of the wide range of crucial homeostatic actions, a suitable amount of body Mg is fundamental for regular cellular and body functioning. Mg disorders have been implicated in the pathogenesis of a large number of diseases, including cardiovascular, metabolic, pulmonary, neurological, and infectious diseases (including COVID-19), bone disease and fractures, and muscular disorders, among others [1]. At least two possible not alternative links (and likely negatively related), i.e., (a) chronic inflammation and (b) decreased immune defense, have been hypothesized to drive the association of a poorly supplied Mg homeostasis and the long list of pathologies that have been associated with a deficient Mg status. It is well established, both in vitro and in vivo, that a Mg deficit and hypomagnesemia result in inflammation and the increased production of free oxygen radicals [1,7,11]. A Mg deficit stimulates the production and release of interleukin-1β and tumor necrosis factor-α, proinflammatory neuropeptides, platelet aggregability and adhesiveness, and lipid peroxidation, while inhibiting endothelial growth and migration and reducing the production of antioxidant hepatic glutathione, superoxide dismutase, and vitamin E [1,12,13,14]. Accordingly, Mg supplementation may significantly reduce different human inflammatory markers, in particular, serum C reactive protein and nitric oxide levels [15].

Mg also stimulates the immune response as it is a co-factor for immunoglobulin (Ig) synthesis, C’3 convertase, immune cell adherence, antibody-dependent cytolysis, IgM lymphocyte binding, macrophage response to lymphokines, and other processes strictly associated with the function of T and B cells [16,17]. In addition, Mg is necessary for the synthesis and activation of vitamin D, another factor involved in the pathogenesis of infectious diseases. Existing data seem to confirm the association between an altered Mg status and several infectious diseases, including COVID-19 [18]. Thus, low serum Mg was found to be associated with a higher incidence of long COVID-19 symptomatology and a significant predictor of mortality, length of stay, and the onset of long COVID-19 symptoms in older persons affected by COVID-19 [19]. In obese COVID-19 patients, reduced renal function and low Mg levels were associated with increased mortality [20]. In over a thousand patients hospitalized for severe COVID-19, the ratio of Mg-to-Ca ≤ 0.20 was strongly associated with mortality [2].

Several studies have implied that Mg plays a role in the development of cardiovascular disease (CVD). An inverse relation has been suggested between Mg intake and hypertension, and hypoMg might be considered a potential risk marker for early CVD [21]. Endothelial function may be compromised under conditions of Mg deficiency, which increases vulnerability to inflammation and the development of arteriosclerosis [22]. HypoMg was suggested to be frequent in patients with atrial fibrillation (AF). The Framingham study showed previously that individuals in the lowest quartile of serum Mg were significantly more likely to develop AF compared to those in the upper quartile. The incidence of hypoMg in patients with de novo AF admitted to the emergency department and intensive care unit was 8.5% [23]. Serum and dietary Mg intake were also associated with markers of subclinical CVD and with the presence of various functional and structural parameters of subclinical CVD [24].

Mg deficit has been associated with an increased risk of psychiatric disorders, including depression, anxiety, and panic attacks, as well as with various neurologic diseases, including cognitive decline and dementia, migraine, and stroke. Mg supplementation may be of help in these conditions and has been used as a coadjutant to prevent migraine attacks and in the treatment of insomnia [25,26,27]. Regarding neuroprotective effects, elevated serum Mg was cross-sectionally associated with greater brain volumes and lower odds of subclinical cerebrovascular disease in participants to the Atherosclerosis Risk in Communities Neurocognitive Study (ARIC-NCS), suggesting beneficial effects on pathways related to neurodegeneration and cerebrovascular damage [28].

A significant body of evidence has suggested that a chronic Mg deficit may be implicated in the pathogenesis of various metabolic disorders, such as metabolic syndrome, insulin resistance, hypertension, dyslipidemia, glucose intolerance, and type 2 diabetes (T2D) [1,29]. The use of Mg supplements may have a beneficial role to improve glucose parameters in people with T2D and in people at high risk of diabetes, reducing plasma glucose per se after a 2 h oral glucose tolerance test and improving insulin sensitivity markers [30].

Mg, because of its mild Ca-channel-blocking properties, has some relaxing effects on bronchial smooth muscle, and a potential use as an adjunct therapy in bronchial constriction conditions has been suggested. Among a group of patients with cystic fibrosis, 47% had hypomagnesemia and 12% insufficient Mg dietary intake; 82% had a high serum Ca/Mg ratio and a low Ca/Mg intake ratio, respectively. Both Ca/Mg ratios were associated with the risk of developing CVD, T2D, metabolic syndrome, and even several cancers. Thus, over half of the patients with cystic fibrosis were at high risk of Mg deficiency and of developing other chronic diseases [31].

Mg is involved in vitamin D and parathyroid hormone (PTH) synthesis and secretion. Mg deprivation is associated with hypoparathyroidism, low production of 1,25-dihydroxyvitamin D3, and resistance to PTH and vitamin D actions [32]. Mg deficiency may contribute to impairing bone mineralization and altering bone strength, quality, and bone mineral density. A systematic review with meta-analysis found a strong association of serum Mg concentrations with incident fractures [33]. Mg supplementation has also been suggested to increase physical performance in relation to the crucial role of Mg to muscle ATP.

In conclusion, numerous human diseases have been connected with Mg deficits, including CVD, hypertension and stroke, cardio-metabolic syndrome and T2D, depression, dementia, digestive and muscular disorders, and bone fragility, among others. Decreased Mg intake in Western countries, due to the wide-spread consumption of ultra-processed food and reduced Mg content in soil, may aggravate the deficit. Maintaining an optimal Mg balance all throughout life may help to maintain a healthy and longer life.
